# Accelerating Earlier Access to Anti-TNF-α Agents with Biosimilar Medicines in the Management of Inflammatory Bowel Disease

**DOI:** 10.3390/jcm14051561

**Published:** 2025-02-26

**Authors:** Gionata Fiorino, Ashwin Ananthakrishnan, Russell D. Cohen, Raymond K. Cross, Parakkal Deepak, Francis A. Farraye, Jonas Halfvarson, A. Hillary Steinhart

**Affiliations:** 1 IBD Unit, San Camillo-Forlanini Hospital, 00152 Rome, Italy; 2 Massachusetts General Hospital, Boston, MA 02114, USA; 3 University of Chicago Medicine, Chicago, IL 60637, USA; 4 The Melissa L. Posner Institute for Digestive Health & Liver Disease at Mercy Medical Center, Baltimore, MD 21202, USA; 5 Division of Gastroenterology, Washington University School of Medicine in St. Louis, St. Louis, MO 63110, USA; 6 Mayo Clinic Florida, Jacksonville, FL 32224, USA; 7 Department of Gastroenterology, Faculty of Medicine and Health, Örebro University, SE-701 82 Örebro, Sweden; 8 Temerty Faculty of Medicine, University of Toronto, Toronto, ON M5S 3H2, Canada

**Keywords:** inflammatory bowel disease, Crohn’s disease, ulcerative colitis, early treatment, anti-TNF-α, biosimilar

## Abstract

Data indicate that earlier initiation of anti-tumor necrosis factor alpha (anti-TNF-α) biologic medicines may prevent progression to irreversible bowel damage and improve outcomes for patients with inflammatory bowel disease (IBD), particularly Crohn’s disease. However, the high cost of such therapies may restrict access and prevent timely treatment of IBD. Biosimilar anti-TNF-α medicines may represent a valuable opportunity for cost savings and optimized patient outcomes by improving access to advanced therapies and allowing earlier anti-TNF-α treatment initiation. Biosimilar anti-TNF-α medicines have been shown to offer consistent therapeutic outcomes to their reference medicines, yet despite entering the IBD treatment armamentarium over 10 years ago, their implementation in clinical practice remains suboptimal. Factors limiting the ‘real’ use of biosimilar anti-TNF-α medicines may include an ongoing lack of understanding and acceptance of biosimilars by both healthcare professionals (HCPs) and patients, as well as systemic factors such as formulary decisions outside of the control of the prescriber. In this review, an expert panel of gastroenterologists discusses HCP-level considerations to improve biosimilar anti-TNF-α utilization in IBD in order to support early anti-TNF-α initiation and maximize patient outcomes.

## 1. Introduction

Inflammatory bowel disease (IBD)—which encompasses Crohn’s disease (CD) and ulcerative colitis (UC)—is a chronic, progressive, immune-mediated gastrointestinal condition characterized by relapses interspersed with periods of remission [1]. As of 2019, there were approximately 4.9 million cases of IBD globally, with an age-standardized prevalence of 59.3/100,000 people [2]. Conventional therapies for IBD include anti-inflammatory aminosalicylates and corticosteroids for mild-to-moderate disease, with immunomodulators, biologic medicines, and novel small molecules for moderate-to-severe disease [3].

The anti-tumor necrosis factor alpha (anti-TNF-α) agents infliximab and adalimumab are effective biologic treatments used to treat people with moderate-to-severe IBD [4,5], as well as populations defined as at ‘high-risk’ for disease complications [6]. TNF-α is a proinflammatory cytokine that contributes to several processes underpinning the pathology of IBD such as mucosal injury and intestinal inflammation [5,7]. However, biologic medicines are associated with a high cost per dose [8,9], and in IBD, much of the substantial economic burden of the disease is driven by the high cost of anti-TNF-α therapies [10,11]. These costs can have implications on treatment accessibility, and in turn, patient outcomes [12,13].

Biosimilar biologics, which are biologics that are designed to have equivalent efficacy and comparable safety to another approved biologic ‘reference’ medicine (Table 1), provide access to effective therapies without inflating costs. Biosimilar medicines provide outcomes consistent with the reference medicine while being typically associated with a lower purchase cost [14,15], thus, offering potential financial savings and increased, affordable access to high-efficacy treatment options [16,17,18]. Biosimilar medicines have been part of the IBD treatment armamentarium for over a decade. As of July 2024, there are 17 biosimilar medicines for IBD approved in the United States (US) and the European Union (EU), and 15 in Canada [18,19,20]. Anti-TNF-α biosimilars currently dominate the IBD biosimilar landscape, but as other high-efficacy treatments begin to reach patent expiry, the IBD biosimilar landscape is expanding with the approval of ustekinumab biosimilars and the development of biosimilars to vedolizumab [18,21,22,23,24,25].

Despite the wide availability of biosimilar anti-TNF-α medicines, their ‘day-to-day’ uptake across the IBD landscape has been suboptimal to date. In this review, an expert panel discusses possible factors inhibiting the ongoing acceptance of anti-TNF-α biosimilars, as well as possible strategies to support their more consistent use to accelerate earlier access to anti-TNF-α agents for management of IBD.

## 2. Methodology

An expert panel of eight gastroenterologists from the EU, US, and Canada met in May and June 2023 to form a global advisory board. The experts were selected by the sponsor based on their expertise in the biological treatment of patients with IBD, as well as broad practical experience and biological treatment integration in their respective geographies. The panel discussed current unmet needs in early, effective IBD management, and proposed strategies to facilitate early anti-TNF-α intervention with respect to potential barriers, based on key themes identified via pre-meeting surveys. Post-event surveys, collated via an online independent platform, consolidated the opinions and insights gathered during the pre-event activities and advisory board meetings. These themes and proposed strategies are outlined in this review.

## 3. The Importance of Timely Treatment in IBD and with Anti-TNF-α Agents

Timely treatment is a relevant consideration in IBD, due to the postulated therapeutic ‘window of opportunity’ whereby treatment initiation early in the disease course can significantly and positively affect disease progression [11]. This is clearest for CD, for which ‘early disease’ is typically classified as ≤18 months following diagnosis [29]. With regard to UC, the evidence supporting the early use of biologic anti-TNF-α agents is less clear, potentially due to a lack of a defined ‘window of opportunity’ for early UC [30,31].

Early use of anti-TNF-α therapies has been shown to improve patient outcomes in CD. The CALM study, for example, demonstrated the importance of timely treatment escalation in maximizing outcomes in patients with active endoscopic CD: patients whose anti-TNF-α therapy was escalated based on clinical symptoms plus biomarkers (tight control) experienced better clinical and endoscopic outcomes than escalations based on symptom-driven decisions alone (clinical management). Compared to the clinical management approach, those following the tight control treatment algorithm received earlier adalimumab treatment and experienced a higher rate of mucosal healing and more positive clinical outcomes [32].

Elsewhere, post hoc analyses of Phase III trials have demonstrated that, in patients with CD treated with anti-TNF-α agents, patients with a shorter disease duration have higher rates of clinical remission versus those with a longer disease duration [33,34]. In another pooled analysis, patients given adalimumab treatment within 1 year of disease duration experienced significantly higher remission rates than those who initiated treatment after the first year of disease [35]. This analysis also showed that early adalimumab initiation led to fewer treatment adjustments, fewer hospitalizations and CD-related surgeries, less activity impairment, and better quality of life [35].

The standard treatment pathway in IBD previously followed a ‘step-up’ approach, whereby aminosalicylates, corticosteroids, and immunomodulators (such as thiopurines or methotrexate) are sequentially tried and ‘failed’ before novel small molecule therapies (e.g., Janus Kinase inhibitors and sphingosine-1-phosphate [S1P] receptor modulators) or biologics are considered. The evidence for early use of advanced therapies in delaying disease progression supports ‘top-down’ or accelerated ‘step-up’ (i.e., combining anti-TNF-α therapies and thiopurines in patients who fail conventional therapy) approaches [11,36,37,38]. The 2024 PROFILE study demonstrated that patients with CD treated according to the ‘step-up’ regimen had a higher rate of urgent abdominal surgeries due to obstructive or penetrating complications compared to those treated in line with the ‘top-down’ approach. Further, patients in the ‘top-down’ group achieved significantly higher rates of steroid-free and surgery-free remission at 1 year than those in the ‘step-up’ group (79% vs. 15%, respectively) [39].

Reflective of the growing evidence base, treatment guidelines are shifting (Table 2); older recommendations describe ‘step-up’ approaches, while more recent guidelines recommend early use of advanced therapies for certain populations, i.e., patients with high-risk disease phenotypes. It is the opinion of this expert panel that people with moderate-to-severe IBD who are at high risk of disease progression and bowel damage, or those who do not respond to conventional therapies within a reasonable time, should be treated as early as possible with advanced therapies such as anti-TNF-α agents to prevent disease progression and mitigate future complications.

## 4. Barriers to Accelerated Anti-TNF-α Use, and the Value of Biosimilar Anti-TNF-α Agents in IBD

One of the main barriers to the earlier use of anti-TNF-α agents is likely cost. Anti-TNF-α therapy has been recognized as a major driver of the economic burden of IBD. The Canadian CD guidelines acknowledge cost as a limiting factor to early biologic therapy, emphasizing the impact of economics on optimized treatment strategies [44]. One large, single-center French study in 2019 reported that anti-TNF-α medication accounted for 84% of the mean annual cost (€15,775) per patient with IBD [47], and a 2024 global survey of 233 physicians involved in IBD care confirmed that the cost of anti-TNF-α biologics limited patient access for 51.9% of respondents [13].

Biosimilar anti-TNF-α medicines have been shown to offer consistent outcomes in terms of clinical efficacy and safety in IBD [48,49], while also permitting substantial cost savings and potential reinvestment. Cost benefits are a key driver of anti-TNF-α biosimilar uptake: in a 2023 global survey of 234 physicians with expertise in IBD, 54.7% of physicians familiar with infliximab and adalimumab biosimilars listed their lower cost as the main reason behind physician decision to use biosimilars in clinical practice [50].

Biosimilar anti-TNF-α agents may permit increased patient access due to their lower cost, which supports the ability to intervene early with highly effective anti-TNF-α agents and target the therapeutic potential of the ‘window of opportunity’ [50,51]. This paradigm has been adopted in other therapy areas; for example, there is promising evidence that biosimilar anti-TNF-α agents have positively impacted patient access to rheumatoid arthritis treatment due to their lower cost [52]. In addition to accelerating earlier treatment initiation, the cost-effectiveness of biosimilars may also enable patients to continue treatment over a longer period to maintain remission and, as such, biosimilar anti-TNF-α medicines may permit IBD treatment continuity [12]. It is important to acknowledge that increased use of infusion anti-TNF-α therapies could impact healthcare costs, necessitating greater infusion service capacity, nursing staff, and infrastructure. However, the availability of both infusion and subcutaneous anti-TNF-α medicines offers treatment flexibility to healthcare systems and a potential strategy to minimize the resource burden associated with infusion-based medicines [53]. And conversely, the earlier intervention enabled by the accessibility of biosimilar anti-TNF-α medicines could help mitigate additional resource demands by reducing other long-term costs associated with disease progression [12]. Further, the potential economic savings afforded by biosimilar anti-TNF-α medicines may allow for wider improvements to IBD care pathways, such as the expansion of healthcare teams, additional support for patients, shortened waiting times, and harmonization of procedures [54].

## 5. Current Attitudes and Potential Barriers Toward Biosimilar Anti-TNF-α Use in IBD

The potential benefits of biosimilar medicines have been discussed in detail in the literature, and numerous professional societies and clinical experts support the use of biosimilar medicines in IBD. The AGA 2019 clinical practice update discussing transitioning to biosimilar medicines when treating IBD states that biosimilars will provide the same effectiveness with no new safety concerns [55]. ECCO (European Crohn’s and Colitis Organisation) endorse transitioning patients from a reference medicine to a biosimilar medicine, while recognizing the ongoing need for outcomes evidence relating to multiple inter-biosimilar transitions [56]. Reflective of this endorsement, a 2023 systematic review showed that 72.2% of ECCO-partnered gastroenterology association guidelines in Europe support the transition of patients with IBD from an anti-TNF-α reference biologic to a biosimilar medicine [57]. Similarly, a 2023 international Delphi consensus of 15 IBD experts reached 100% agreement on the notion that the transition from a biologic to a biosimilar medicine is safe and effective [50].

With regard to anti-TNF-α biosimilars specifically, an Italian Delphi consensus statement published in 2023 agreed that the cost-efficacy profile of anti-TNF-α biosimilars warrants their first-line use in IBD [58]. In a separate 2023 statement from 18 gastroenterology associations in the European Economic Area, 14 (77.8%) endorsed initiating anti-TNF-α therapy with a biosimilar for the treatment of IBD [57]. Finally, in a third 2023 statement, 15 IBD experts from 13 countries agreed that anti-TNF-α biosimilars can be used both in biologic-naïve patients and those already treated with the reference medicine; transitioning from a reference anti-TNF-α biologic to a biosimilar counterpart can be performed at any time; and switching to biosimilar anti-TNF-α agents is a way to reduce the costs associated with advanced therapies and to increase treatment accessibility [50].

At the healthcare professional (HCP) level, an ECCO survey of IBD specialists (N = 118) conducted in 2015 indicated that only 19.5% of respondents had little or no confidence in the use of biosimilars—a decrease from 63% in the 2013 ECCO survey [59]. Similarly, 44.4% of respondents believed that biosimilar medicines could be used interchangeably with the reference, an increase of 38.6% from 2013 [59]. A survey of 233 physicians from 63 countries conducted in 2023 revealed that 26.6% of responders believed additional research or evidence was not needed to support the use of biosimilars in IBD, indicating a nascent level of biosimilar confidence among HCPs [13].

## 6. Exploring the Disconnect Between the Guidance and Real Use of Biosimilar Anti-TNF-α Medicines in Clinical Practice

Thus, based on the available guidance and survey data, there should be few barriers to biosimilar anti-TNF-α agents being consistently used to support high-efficacy treatment goals in IBD. Yet in the experience of the expert panel, despite the need for cost-effective anti-TNF-α treatments in IBD, supporting evidence for consistent outcomes, and endorsement from guiding bodies, the uptake of biosimilar anti-TNF-α medicines in practice has remained suboptimal. Below are discussed factors that the panel believe may have limited the use of biosimilar anti-TNF-α medicines in IBD to date, with strategic suggestions to mitigate these potential ‘barriers’ and support accelerated use of anti-TNF-α therapy (summarized in Table 3).

### 6.1. Barrier #1: Complex Reimbursement/Insurance Policies

**THE BARRIER(s):** As with all advanced therapies for IBD, a fundamental barrier to biosimilar anti-TNF-α implementation identified by the panel is complex reimbursement and insurance policies. For example, the Czech Republic, France, Germany, Romania, Spain, and Sweden generally require failure or intolerance of one non-biologic treatment before reimbursable biologic treatment is permitted; other European countries such as Hungary, Latvia, Poland, and Slovakia require failure of two non-biologic treatments before patients with CD are eligible for biologic therapy [60]. ‘Too strict reimbursement criteria’ and the ‘lengthy authorization process’ are noted barriers to physicians prescribing early biologic treatment for CD [60]. Additionally, there are some data to suggest that inefficient purchasing procedures may also limit effective implementation of anti-TNF-α biosimilars in practice [61].

Similar issues have been noted in Canada, where the permitted first-line use of biologic therapy varies across provinces and is dependent on public drug coverage [62]. In many cases, biologics are effectively positioned as third-line therapies [38]. However, some provinces have recently approved access to biosimilar anti-TNF-α agents and vedolizumab without the requirement for loss of efficacy of an immunomodulator [62], indicating a potential shift towards the ‘top-down’ approach. Criteria for reimbursement in Canada may vary widely for patients with private drug insurance and are dependent on the insurance provider; for example, a single-center study from Ontario showed that patients with public drug insurance experienced greater delay in first dose administration of anti-TNF-α therapy compared to those with private insurance (53 vs. 34 days, respectively) [63].

The barrier associated with reimbursement policies is particularly true for the US, where many insurance companies require prior authorization for certain medicines—including biologic medicines—which may result in delays in care due to the associated administrative work. Furthermore, many insurance companies consider biologic therapies as ‘specialty drugs’, and implement patient co-pays, which may result in increased out-of-pocket spending for patients compared to medicines that are not considered ‘specialty drugs’ [64]. Of 34 US-based insurance policies analyzed in a 2022 study, 91% generally required failure of at least one conventional therapy for both CD and UC before permitting biologic treatment; only 14.7% and 17.7% permitted the first-line use of biologics for CD and UC, respectively [65]. While not explicitly stated, but likely inferred, the reason for non-compliance of US insurance companies with IBD guidelines is cost [11]. In addition, Pharmacy Benefit Managers often receive higher rebates for placing higher-cost reference medicines on formularies, creating a financial incentive to favor reference anti-TNF-α biologics over their less expensive biosimilar counterparts [51]. Panel members from the US note that prescribing decisions are mandated by the payer, and HCPs may not actively be permitted to choose between a reference biologic or biosimilar anti-TNF-α agent.

**POTENTIAL SOLUTIONS:** The panel recognizes that reimbursement issues may be systemic and are outside of the control of the individual prescriber to resolve. However, the removal of the payer requirement for patients to first fail on traditional therapies, some of which are used off-label, would allow for the early initiation of biologic and biosimilar anti-TNF-α therapies. This change would allow for the ‘top-down’ treatment paradigm to be effectively implemented into clinical practice (while adhering to the indications of available treatments) and potentially support a reduction in the cumulative costs of sustained disease.

### 6.2. Barrier #2: HCP Skepticism Towards Biosimilar Medicines

**THE BARRIER:** As noted in Section 5, positive advancements in HCP attitudes toward biosimilars are evident [59], but some resistance to their uptake persists. In a US study from 2020 assessing physician willingness to transition from reference to biosimilar infliximab, 72.8% refrained from prescribing any biosimilar, while 3.5% prescribed only the biosimilar [66].

Skepticism has been reported in some regions surrounding biosimilars’ comparability to their reference medicines, which may be a source of hesitancy for HCPs to prescribe, or for patients to receive a biosimilar therapy [67,68]. This skepticism may be due to developmental differences between biosimilar and de novo biologic medicines in terms of clinical trial design and evidential requirements, as well as a lack of understanding around concepts such as extrapolation and authorization of indications [67,68,69].

Insufficient understanding of biosimilar transitioning data may lead to a lack of confidence, or hesitancy as to whether a reference medicine may truly be safely substituted for a biosimilar, ultimately impacting their utilization in clinical practice. In addition, the definition of terminologies associated with biosimilar medicines—for example, ‘interchangeability’—can vary across geographies, potentially causing confusion. Once approved in the EU, a biosimilar can be considered ‘interchangeable’ with its reference medicine [70]. In the US and some Canadian provinces, however, a biosimilar may also be given a formal ‘interchangeable’ definition [17,71,72]. This primarily impacts how a biosimilar may be substituted at the pharmacy level but may also impact HCP trust in a biosimilar medicine that does not have this designation.

It is critical to instill the same level of trust in the process of biosimilar development and regulatory approval as there is for reference medicines amongst the HCP community. Patients may internalize any skepticism held by their HCP about biosimilar medicines, potentially affecting their confidence in, and willingness to receive, such therapies [73]. The expert panel strongly supports the assertion that an individual may be transitioned from a reference medicine to an approved biosimilar medicine at any point, without impacting that individual’s ongoing treatment outcomes. The members of the panel agree that key clinical criteria that support a transition in IBD are as follows:

(1) A lack of clinical need to restrict biosimilars to biologic-naïve patients;
(2) The absence of limitations or concerns about effectiveness or safety;
(3) Patients with evidence of remission with improvement or normalization of biomarkers or endoscopy, which are often regarded as the ‘best candidates’ for a biosimilar transition.

**POTENTIAL SOLUTIONS:** The acceptance and utilization of biosimilars in IBD, and consequent improved access to early anti-TNF-α therapy, can be improved by continued education on the robustness of the biosimilar development process and the safety of transitioning between reference and biosimilar medicines [9]. As outlined by the most recent ECCO survey in 2015, there may be a need for more clear and balanced biosimilar education: Compared with the 2013 ECCO survey results, the 2015 survey reported an increase in HCPs reporting ‘the need to promote and inform about biosimilars’, from 65.7% up to 75.2% [59]. However, while 56% of respondents found biosimilar-focused educational activities ‘fair and adequate’, 15.3% of respondents considered them ‘confusing’, and 12.7% of respondents believed the education to be ‘too optimistic about efficacy and safety’ [59]. As more biosimilars enter the IBD landscape, there is a pressing need for targeted educational efforts within the HCP community to enhance understanding, reduce confusion, and consolidate trust in these therapies. Key strategies for improving HCP education and, in turn, encouraging biosimilar uptake were outlined by Oskouei and Kusmierczyk in 2021 [73]. Suggested tactics include:

(a) Continued research through randomized clinical trials, long-term data, and real-world evidence to address HCP concerns about the safety, efficacy, and immunogenicity of biosimilar medicines;
(b) Developing tailored biosimilar educational materials that address the specific needs and concerns of different HCP sub-populations, recognizing that biosimilar education should not be ‘one-size-fits-all’;
(c) Integrating biosimilar education into medical and pharmacy school curriculums to build a strong knowledge foundation.

Other strategies to build HCP trust in biosimilar medicines and improve their uptake include encouraging peer-to-peer education to share real-life experiences with biosimilar medicines; engagement with and guidance from professional medical societies; and using various media channels to facilitate educational initiatives [73,74]. Members of the expert panel from EU regions noted that when biosimilar medicines first became available, experience-sharing among colleagues and visiting centers with experience in biosimilars was effective in reassuring and convincing HCPs of their use.

### 6.3. Barrier #3: Patient Concerns About Advanced Therapy and Biosimilar Medicines

**THE BARRIER(s):** Patient concerns surrounding advanced therapies, such as anti-TNF-α agents, may lead to reluctance to begin treatment and also hinder the uptake of anti-TNF-α biosimilar medicines. Previous data have shown that patients with IBD may be reluctant to receive advanced biologic therapy, perceiving them as ‘risky’ and ‘dreadful’ (defined as how scary, uncontrollable, fatal, or catastrophic the exposure is perceived to be) [75]. Social media data show that people with IBD frequently engage in discussions about negative experiences surrounding biologic therapies, expressing concerns about side effects, hesitation to initiate, and treatment discontinuation [76]. Anecdotal data from the US highlights ‘patient fear of biologics’ as a key reason for delayed biologic initiation in IBD [77].

Additionally, anti-TNF-α agents for IBD are given by intravenous or subcutaneous injection, and some patients prefer receiving an oral, non-injectable medicine [78]. Patient questionnaires indicate that a substantial cause of the fear towards biologic therapies is the injection/infusion aspect, with past negative experiences (e.g., injection site reactions and pain) or fear of needles potentially contributing to treatment hesitancy [79,80]. Biosimilar anti-TNF-α agents, which match the posology and administration of their reference medicines [16,81], are likely affected by these issues, and such issues should be considered when counseling a patient on a reference or biosimilar biologic therapy.

People with IBD may have pre-existing negative attitudes about the perceived risks of anti-TNF-α agents that may then be combined with a lack of knowledge and trust toward biosimilar medicines. This lack of biosimilar understanding by patients is evident across both North America and the EU. An online survey conducted by the European Federation of Crohn’s and Ulcerative Colitis Associations published in 2017 found that 62% of patients with IBD had not heard of biosimilars, and those who did were concerned about their safety profile and effectiveness [82]. In addition, only 31% of respondents stated they would be fully confident about a biosimilar medication, even if prescribed and explained by a treating physician [82]. A large 2017 focus group project carried out by five Canadian patient organizations (including Crohn’s and Colitis Canada and the Gastrointestinal Society) reported that people with inflammatory diseases were hesitant to receive biosimilar medicines because they believed them to be unsafe, or of lesser quality than the reference biologic. Furthermore, some participants felt uncomfortable answering questions related to biosimilar medicines because they felt they did not know enough about them to make an informed response [83].

In a 2017 US single-center survey of 121 people with IBD, 57% of respondents felt uncomfortable exchanging their current medication for a biosimilar, and 76% felt uncomfortable using a biosimilar medicine that had not been tested specifically for CD or UC [84]. A US survey among 500 people with IBD published in 2023 revealed that this hesitancy persists: 66% of respondents had not heard of biosimilars and only 43% of biologic users would accept switching to a biosimilar [85]. Reasons for not wanting to accept a switch included concerns about side effects, financial support (such as insurance coverage and co-pay assistance), and the assumed efficacy of the biosimilar medicine [85].

**POTENTIAL SOLUTIONS:** The expert panel notes that insufficient patient understanding, trust, and acceptance of biosimilar medicines remains a highly influential barrier to improving biosimilar anti-TNF-α use and early treatment intervention in IBD. Efforts to empower patients to seek effective support for their IBD have been made, e.g., numerous non-profit organizations and IBD support groups have implemented outreach programs to cultivate a patient community and apply improvements to clinical practice and outcomes [64]. The European Federation of Crohn’s and Ulcerative Colitis Associations (EFCCA) Patient Guidelines include comprehensive explanations surrounding various treatment options, including anti-TNF-α agents, and aim to facilitate patient education and empowerment throughout their disease journeys [86]. Such initiatives help patients to be appropriately informed about all possible treatment options and should be leveraged to increase patient confidence in advanced therapies, such as anti-TNF-α agents.

With specific regard to acceptance of biosimilar medicines, five essential aspects of patient communication have been previously proposed [87]:

(a) Provide understandable and up-to-date information, using simple language and avoiding medical jargon;
(b) Communicate openly, positively, and transparently to promote biosimilar acceptance and reduce the likelihood of a nocebo effect;
(c) Tailor information and communication styles to individual patient needs;
(d) Communicate with ‘one voice’ to ensure consistent communication across all stakeholders;
(e) Use supportive audiovisual material to enhance understanding.

**Figure 1:**
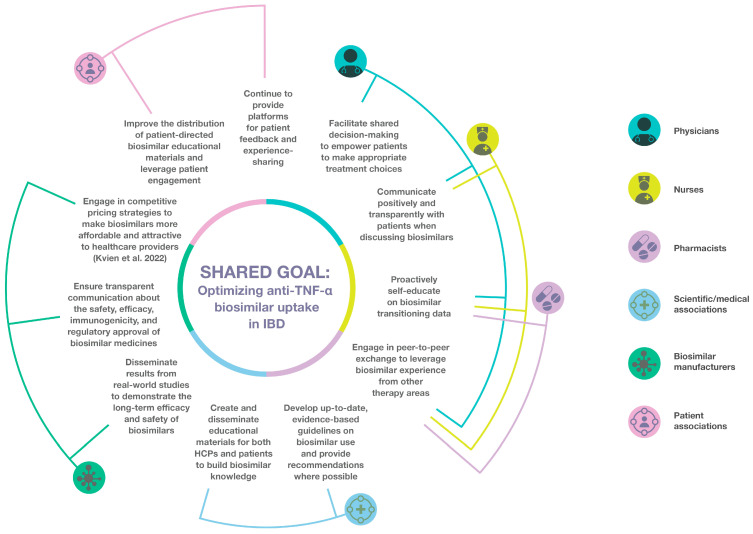
Conceptual framework for optimizing biosimilar anti-TNF-α use in IBD. HCP, healthcare professional; IBD, inflammatory bowel disease; TNF-α, tumor necrosis factor alpha [51].

All healthcare providers, both at the patient (physician, pharmacist, nurse) and organizational (scientific/medical association, regulatory authority, patient organization) level should adopt such strategies to ensure accurate and consistent information is provided to patients about biosimilar medicines and how a biosimilar can support treatment goals. As noted above, avoidance of a nocebo effect, whereby a patient experiences negative outcomes unrelated to the treatment’s inherent pharmacologic effects, is a key consideration for biosimilar anti-TNF-α acceptance [67,88]. A nocebo effect can occur in any medical setting, triggered by negative expectations about the treatment, poor patient–physician communication, or gaps in awareness [67], and has previously been observed in IBD biosimilar switching studies [89,90]. Figure 1 provides a proposed ‘conceptual framework’ that highlights the role of each HCP in educating and supporting peers and patients to accelerate the use of biosimilar anti-TNF-α medicines in IBD.

## 7. Conclusions

It is the opinion of this expert panel that better utilization of biosimilar anti-TNF-α agents is key for accelerating and optimizing early, high efficacy treatment in IBD, particularly for patients with moderate-to-severe CD. Extensive real-world evidence demonstrates that biosimilar anti-TNF-α medicines can offer substantial benefits to both patients with IBD and healthcare systems, and the change in regional and international guidelines reflects a broader paradigm shift to maximize benefits via ‘top-down’ treatment approaches, particularly for moderate-to-severe disease.

The panel acknowledges that the barriers discussed here represent a European and North American rather than global perspective. Treatment approaches and cost considerations associated with the availability and use of biologic and biosimilar medicines can vary substantially by country. In regions with limited access to advanced therapies, the availability of cost-effective biosimilar medicines may have an even more critical role in improving patient outcomes in IBD. In Europe and North America, outside of the influence of systemic structural barriers such as complex reimbursement and insurance policies, there are HCP-level actions that can be made now to support better use of biosimilar anti-TNF-α medicines in early IBD treatment and, thus, accelerated treatment initiation and long-term optimized outcomes.

Relevant, accessible education—for patients and practitioners—must be prioritized. Educational efforts from pharmaceutical providers as well as peer-to-peer sharing should target all areas of the multidisciplinary team in IBD to support confidence in the concept and clinical suitability of biosimilar anti-TNF-α medicines and be able to assuredly convey this information to patients to support successful treatment outcomes. By implementing such strategies, we can better leverage the benefits of biosimilar anti-TNF-α medicines and better support the ‘top-down’ approach to IBD treatment, ultimately improving long-term disease management and patient outcomes, as well as paving the way for the future biosimilar landscape in IBD.

## Figures and Tables

**Table 1:** Differentiation between biologic and biosimilar medicines.

Term	Definition
**Biologic medicine**	Biologic medicines are large, complex molecules that contain active substances derived from living sources such as cells or organisms [16,26]. Classes of biologic medicines commonly used to treat IBD include anti-TNF-α agents (e.g., infliximab, adalimumab, golimumab, and certolizumab), adhesion molecule antagonists (e.g., vedolizumab), interleukin antagonists (e.g., ustekinumab), and JAK inhibitors (e.g., tofacitinib) [27].
**Biosimilar medicine**	A biosimilar is a biologic medicine that is designed to match an approved biologic medicine (the ‘reference’ medicine) in structure and function. Biosimilar medicines are reviewed and approved based on a rigorous ‘totality of evidence’ approach, to confirm that there are no clinically meaningful differences in safety, efficacy, and immunogenicity from the reference medicine [16,26,28]. Biosimilar medicines used to treat IBD include biosimilars of infliximab, adalimumab, and ustekinumab biologics [18,19,20].

IBD, inflammatory bowel disease; JAK, Janus kinase; TNF-α, tumor necrosis factor alpha.

**Table 2:** Summary of current guidelines on the early use of anti-TNF-α therapy in IBD.

**Professional Society**	**IBD**	
International Organization for the Study of IBD (2021) [40]	STRIDE initiative recommends a treat-to-target approach, whereby treatment is delivered early and intensely to optimize patient outcomes	
**Professional Society**	**CD**	**UC**
American Gastroenterological Association (2021) [36,41]	Recommends anti-TNF-α therapies over no treatment for inducing and maintaining remission for luminal disease Acknowledges that the ‘step-up’ approach for moderate-to-severe CD “may result in clinical harm from delaying appropriate disease treatment”	Recommends the use of anti-TNF-α therapies over no treatment for moderate-to-severe UC; conditionally recommends early anti-TNF-α use after failure of 5-aminosalicylate therapy Acknowledges that delayed treatment in patients at high risk of colectomy “may be harmful due to ongoing active disease, increasing risk of UC-related complications, hospitalization, colectomy, and overall inferior quality of life”
European Crohn’s and Colitis Organisation (2020) [42,43]	Acknowledges that, from post hoc analyses of clinical trials, the early initiation of anti-TNF-α agents may enhance efficacy, induce and maintain clinical remission, and prevent complications	Recognizes that there is less evidence for UC than CD for the impact of early treatment initiation and escalation, but the conventional approach to treating UC is becoming outdated in a subset of patients Notes the optimal time point for the introduction of anti-TNF-α therapy in UC is yet to be defined
Canadian Association of Gastroenterology (2019) [44,45]	Recommends anti-TNF-α agents for moderate-to-severe CD after conventional therapy of corticosteroids, thiopurines, or methotrexate fail to achieve complete remission Recognizes the ‘top-down’, early anti-TNF-α approach but reserve it for high-risk patients or those with a poor prognosis, due to the cost of such therapies	Recommends anti-TNF-α therapy only in those who have failed prior thiopurine or corticosteroid therapy Early initiation of anti-TNF-α agents in a ‘top-down’ approach is not discussed
American College of Gastroenterology (2018) [37,46]	Recommends anti-TNF-α agents for moderate-to-severe CD after corticosteroids, thiopurines, or methotrexate failure	Recommends anti-TNF-α therapy for the induction of remission of moderately-to-severely active UC Acknowledges a lack of head-to-head data between the ‘top-down’ and ‘step-up’ algorithms

CD, Crohn’s disease; IBD, inflammatory bowel disease; TNF-α, tumor necrosis factor alpha; UC, ulcerative colitis.

**Table 3:** Potential barriers and strategies to optimize biosimilar anti-TNF-α use and facilitate accelerated intervention.

Potential Barrier	Solution-Based Strategy
**The impact of reimbursement and insurance policies**	Optimize referral networks Support initiatives that challenge unnecessary practice burdens Remove the payer requirement for patients to fail traditional therapies, enabling earlier biologic initiation
**Persisting HCP misconceptions and lack of education around biosimilar medicines**	Provide education to: o Instill HCP trust in the biosimilar development and regulatory approval processes o Alleviate misconceptions around biosimilar efficacy and safety o Empower confident patient communications
**Patient fear and concerns surrounding biologic medicines**	Empower patients to seek effective support for their IBD and build confidence in their understanding of advanced therapies Provide patient-directed educational efforts to allow patients to be fully informed on available treatment options Offer tailored, sensitive education conveyed in a way that avoids precipitating the nocebo effect

HCP, healthcare professional; IBD, inflammatory bowel disease; TNF-α, tumor necrosis factor alpha.

## Data Availability

Not applicable.
